# Soil Macroinvertebrate Presence Alters Microbial Community Composition and Activity in the Rhizosphere

**DOI:** 10.3389/fmicb.2019.00256

**Published:** 2019-02-22

**Authors:** Natalie Bray, Jenny Kao-Kniffin, Serita D. Frey, Timothy Fahey, Kyle Wickings

**Affiliations:** ^1^Department of Entolomology, Cornell AgriTech, Cornell University, Geneva, NY, United States; ^2^School of Integrative Plant Science, Cornell University, Ithaca, NY, United States; ^3^Department of Natural Resources and the Environment, University of New Hampshire, Durham, NH, United States; ^4^Department of Natural Resources, Cornell University, Ithaca, NY, United States

**Keywords:** soil macroinvertebrates, soil microbial communities, rhizosphere, mesocosms, microbial activity

## Abstract

Despite decades of research, our understanding of the importance of invertebrates for soil biogeochemical processes remains incomplete. This is especially true when considering soil invertebrate effects mediated through their interactions with soil microbes. The aim of this study was to elucidate how soil macroinvertebrates affect soil microbial community composition and function within the root zone of a managed grass system. We conducted a 2-year field mesocosm study in which soil macroinvertebrate communities were manipulated through size-based exclusion and tracked changes in microbial community composition, diversity, biomass and activity to quantify macroinvertebrate-driven effects on microbial communities and their functions within the rhizosphere. The presence of soil macroinvertebrates created distinct microbial communities and altered both microbial biomass and function. Soil macroinvertebrates increased bacterial diversity and fungal biomass, as well as increased phenol oxidase and glucosidase activities, which are important in the degradation of organic matter. Macroinvertebrates also caused distinct shifts in the relative abundance of different bacterial phyla. Our findings indicate that within the rhizosphere, macroinvertebrates have a stimulatory effect on microbial communities and processes, possibly due to low-intensity microbial grazing or through the dispersal of microbial cells and spores by mobile invertebrates. Our results suggest that macroinvertebrate activity can be an important control on microbially-mediated processes in the rhizosphere such as nitrogen mineralization and soil organic matter formation.

## Introduction

Soil microbes are recognized as the main drivers of soil organic matter (SOM) formation and decomposition. However, soil invertebrates can also directly impact SOM processes, particularly through litter decomposition (Coleman, 2008). Soil macroinvertebrates, including earthworms, soil-dwelling insects, myriapods and isopods, are categorized by their larger size (>2 mm) and are known for their roles as ecosystem engineers where they modify soil habitat as well as resource distribution (Jones et al., 1996). This invertebrate group is functionally distinct from other soil biota such as mesofauna (0.1–2 mm) and microfauna (<0.1 mm) (Swift et al., 1979; Beare et al., 1995; Brussaard, 1997; Coleman et al., 2004; Bradford et al., 2007) and through the transport and breakdown of plant litter they are capable of accelerating the incorporation of plant residues into soil (Bradford et al., 2002; Wall et al., 2008; Garcia-Palacios et al., 2013).

While the direct impact of soil macroinvertebrates on soil carbon cycling is notable, it has been proposed that the greatest contribution invertebrates make to soil processes is through their interactions with the soil microbial community (Grandy et al., 2016; Trap et al., 2016). Soil invertebrate-microbe interactions can take diverse direct and indirect forms. For instance, grazing, by macroinvertebrates, such as isopods and millipedes, can reduce fungal biomass (Crowther et al., 2011a), alter fungal community composition (Crowther et al., 2011b) and increase fungal extracellular enzymatic activity (Crowther et al., 2011c). Microbial grazing by macroinvertebrates has also been shown to increase bacterial biomass (Lussenhop, 1992; Dempsey et al., 2013). In addition to affecting microbial communities directly, macroinvertebrates also impact soil microbes indirectly by altering the composition and distribution of microbial resources. For instance, through litter fragmentation and translocation between litter and soil layers, earthworms and millipedes can alter microbial biomass (Maraun and Scheu, 1996; Chang et al., 2017) and community composition (Dempsey et al., 2011, 2013). Furthermore, macroinvertebrates can alter litter chemistry through ingestion and gut passage, changing resource availability and quality for microbes (Filley et al., 2008). These examples illustrate that macroinvertebrates can alter microbial communities through diverse channels and suggest that such alterations may have important consequences for key belowground SOM processes.

There has been a long-standing interest in quantifying the effects of macroinvertebrates on soil microbial dynamics. To date, studies have focused primarily on microbial responses to single species of invertebrates, with the majority of studies focusing on earthworms, isopods or millipedes (Maraun and Scheu, 1996; Groffman et al., 2004; McLean et al., 2006; Crowther et al., 2011a,b,c, 2015; Dempsey et al., 2011). Studies attempting to link macroinvertebrate-driven changes in microbial community composition to shifts in microbial function have highlighted that macroinvertebrates induce strong changes in microbial biomass and can elicit contrasting responses in fungi and bacteria (Dempsey et al., 2013; Chang et al., 2017). However, under natural conditions, microbial communities are exposed simultaneously to diverse macroinvertebrate taxa, each with its own potential to impact microbial communities through distinct pathways. Thus, in order to fully understand the role of macroinvertebrates in shaping soil microbial dynamics in natural settings it will be important to quantify microbial responses to mixed macroinvertebrate communities, which are likely have multiple interactive effects on microbes.

Previous efforts to quantify the impact of soil invertebrates on soil microbial community composition and function have also focused almost exclusively on leaf litter and other surface-confined plant residues as the dominant microbial resource input (e.g., Bradford et al., 2002; Bardgett, 2005; Hättenschwiler et al., 2005), and much less is known about how invertebrates influence microbial interactions with root-derived organic matter. Root-derived carbon differs greatly from foliar litter in both quantity and chemistry (Gale et al., 2000; Jones et al., 2009; Bradford et al., 2012) and it is acknowledged as a major resource for soil food webs (Albers et al., 2006; Pollierer et al., 2007; Elfstrand et al., 2008). Recent studies have also demonstrated that root-derived inputs constitute the dominant source of SOM (Austin et al., 2017; Shi et al., 2018; Sokol et al., 2019). Given the growing awareness of the importance root-derived inputs for SOM formation, and the acknowledged role of soil microbes in driving SOM formation (Cotrufo et al., 2013; Wieder et al., 2014), it is equally important to quantify the effects of soil animals on rhizosphere microbial communities.

The goal of this study was to better understand the impacts of macroinvertebrates on microbial community composition and function in the rhizosphere. We selected an urban grass ecosystem where rhizosphere inputs are important for soil biological communities (De Deyn et al., 2003; Ostle et al., 2007), where soil invertebrates are abundant and diverse (Rochefort et al., 2006; Pouyat et al., 2010) and where SOM cycling and accrual are of particular interest for soil carbon management (Qian and Follett, 2012; Shi et al., 2012). In order to quantify the importance of soil macroinvertebrates as mediators of soil microbial function, we carried out an exclusion-based mesocosm study within the root zone of lawn-type grasses and tracked changes in microbial community composition and activity over 2 years. Because macroinvertebrates are known to graze intensively on fungal biomass, we hypothesized that their inclusion would (1) decrease fungal biomass but stimulate bacterial biomass; (2) decrease fungal diversity and increase bacterial diversity, and overall, stimulate the activity of extracellular enzymes and subsequently C and N mineralization rates. Finally, we anticipated that the above responses to macroinvertebrates would change over the course of the 2- year experiment.

## Materials and Methods

### Research Site and Experimental Design

This experiment was conducted at the Bluegrass Lane Turf and Landscape Research Center (Ithaca, NY, United States) from 2015 to 2017 in an area dominated by turf-type tall fescue (*Festuca arundinacea*) and regularly mowed to a height of approximately 7.5 centimeters. Soils at the site are classified as Arkport fine sandy loam (mesic Lamellic Hapludalfs) with a pH of 6.5. Total annual precipitation at the site was 80.8 cm in 2016 and 108.1 cm in 2017 (Northeast Regional Climate Center, 2016–2017). In the fall of 2015, soil mesocosms were installed in 16 replicate 1 m^2^ plots for the purpose of manipulating soil faunal communities. Thirty-two mesocosms (10 cm height × 10 cm in diameter) were created using stainless steel mesh: 16 with 5 mm openings to allow for colonization by both soil macroinvertebrates and mesoinvertebrates (“macro-mesh”) and 16 with 1 mm openings to restrict colonization by macroinvertebrates (“meso-mesh”). Both mesocosm types allowed for ingrowth of grass roots. All mesocosms were filled with defaunated soils that were collected as one intact core per mesocosm (10 cm depth × 10 cm in diameter) and each core was kept separate throughout the defaunation process. These soils were subsequently sieved (4 mm) then subjected to two heating and freezing cycles (24 h at +80°C, 24 h at -20°C) to eliminate soil fauna (adapted from Huhta et al., 1989; Bardgett et al., 1998). Soils were then adjusted to field moist conditions (based on gravimetric moisture content, recorded at the time of soil collection) and added to mesocosms at about field bulk density. The mesocosms then installed to 10 × 10 cm core holes and buried under an actively growing grass layer (2–3 cm thick) by replacing a live turfgrass plug directly above each newly installed mesocosm. The placement of the live turfgrass plug was done to ensure root growth directly into the mesocosms to mirror the extensive root growth in the grass system. Two mesocosms were placed in adjacent 1 m^2^ plot in November 2015 and all mesocosms were in a uniform 24 m^2^ area. In November 2016, 1 year after burial, 16 mesocosms (8 macro-mesh and 8 meso-mesh) were harvested and the remaining mesocosms were harvested in November 2017, 2 years after burial.

Soils were carefully removed from the mesocosms in the lab and divided for soil fauna extractions, soil microbial community analyses and analysis of physical and chemical properties. Soils for microbial analysis were sieved (4 mm). Soils were subsampled for extracellular enzyme activity, microbial biomass through phospholipid fatty acids (PLFAs), bacterial and fungal community composition and diversity and stored at -20°C. Soils for analysis of physical and chemical properties were air-dried and stored at ambient temperature. Ingrown roots within each mesocosm from the turf layer directly above were carefully separated, dried and weighed. Soil samples were also collected directly adjacent to the mesocosms prior to each mesocosm harvest and handled and analyzed in the same way to assess mesocosm effects.

### Soil Invertebrates

All macroinvertebrates from harvested mesocosms were collected by hand, counted and identified to major taxonomic groups. A subsample (approximately 150 g) of soil was placed onto modified Berlese funnels for heat extraction of soil mesofauna. Extraction temperature began at 30°C and was increased by 10°C over a 3 day period, to a final extraction temperature of 50°C. Soil invertebrates were identified to major taxonomic groups using Borror and Delong Key for Insects (Triplehorn and Johnson, 2005) for insects and collembola and following the taxonomy in the Key to Major Mite Taxa (Walter, 2005) for mites. Abundances are reported as number of individuals kg^-1^ dry soil.

### Microbial Community Composition and Diversity

DNA was isolated from a 250 mg soil subsample using the PowerSoil DNA Isolation Kit (Mo Bio Laboratories, Inc., Carlsbad, CA, United States). For 16S rRNA amplifications, 1 μl of each bacterial primer and 8 μl of 5 PRIME HotMaster Mix (5 PRIME, Inc., Gaithersburg, MD, United States) were added to 1:10 diluted DNA solutions, yielding 20 μL reaction volumes. The universal bacterial primers 341F (5′-195 CCTACGGGNGGCWGCAG-3′) and 805R (5′-GACTACHVGGGTATCTAATCC-3′) (Herlemann et al., 2011) were used with overhangs included for index attachment. The PCR protocol for 16S rRNA gene amplifications was as follows: 94°C for 2 min; 25 cycles of 94°C for 20 s, 55°C for 20 s and 72°C for 30 s; with a final elongation at 72°C for 5 min, using a Bio-Rad C1000 Thermal Cycler (Bio-Rad, Hercules, CA, United States). For fungal internal transcriber spacer (ITS) amplifications, 0.5 μl of each primer, 8 μl of 5 prime HotMaster mix, and 1 μl DMSO were added to 1:10 diluted DNA solutions. For ITS amplifications, we used the primers ITS1F (5′-CTTGGTCATTTAGAGGAAGTAA-3′) and 58A2R (5′-CTGCGTTCTTCATCGAT-3′) (Gardes and Bruns, 1993; Martin and Rygiewicz, 2005) with the required adaptors attached. The PCR settings for ITS were as follows: 94°C for 3 min; 35 cycles of 94°C for 20 s, 45°C for 30 s and 72°C for 45 s; with a final elongation at 72°C for 5 min, and were conducted on the Bio-Rad C1000 Thermal Cycler.

The amplicons were cleaned with MagBio HighPrep PCR beads (MagBio Genomics, Gaithersburg MD, United States) in clear 96-well plates. The cleaned amplicons received attachments of unique two-barcode index combinations through combination of the following into each well of a 96-well plate: 5 μL of sample, 2.5 μL of forward and reverse primers containing designated barcodes that target the attached overhangs, 2.5 μL of water, and 12.5 μL of Q5 High Fidelity 2X Master Mix (New England Biolabs, Inc., Ipswich, MA, United States). The PCR conditions for index attachment were set as: 98°C for 1 min; 8 cycles of 98°C for 15 s, 55°C for 30 s and 72°C for 20 s; with a final elongation at 72°C for 3 min. The resulting barcoded amplicons were normalized by using the SequalPrep Normalization Kit (Thermo Fisher Scientific, Waltham, MA, United States). A 5 μL aliquot of each normalized sample was added into separate pools for 16S rRNA gene and ITS amplicons. The mixture was concentrated and run on 1.2% agarose gels, and bands of the expected size were excised and processed using the Promega Wizard SV Gel and PCR Clean-Up System (Promega, Middleton, WI, United States) to a final concentration of 30 μL. Samples were sequenced on the Illumina MiSeq at the Cornell Genomics Facility (Ithaca, NY, United States), using a 500-cycle MiSeq Reagent Kit v.2 for the ITS pool, and a 600-cycle MiSeq Reagent Kit v.3 for the 16S rRNA gene pool.

Initial sequence processing was based on the Brazilian Microbiome Project Pipeline (Pylro et al., 2014), with some modifications (see Howard et al., 2017). In mothur v.1.35.1 (Schloss et al., 2009), paired-end sequences were merged (make.contigs), primers trimmed (trim.seqs, pdiffs = 2, maxambig = 0), and singleton sequences removed (unique.seqs - > split.abund, cutoff = 1). In QIIME (Caporaso et al., 2010), clustering of 97% OTUs and chimera removal (RDP Gold and UNITE databases provided by http://www.brmicrobiome.org/) were performed using VSEARCH v.2.82 (Rognes et al., 2016). In mothur, representative OTU sequences were classified (classify.seqs, cutoff = 80) using the GreenGenes v. 13.8 database for 16S rRNA gene sequences and UNITE v.7 database for ITS sequences, and OTUs that were suspected to not be of fungal or bacterial origin were removed (remove.lineage). OTU tables were formatted by QIIME. The number of 16S rRNA gene sequences and ITS sequences were rarefied based off of the minimum available reads per soil sample to normalize inter-sample comparisons for downstream analyses in R. All raw sequencing files were submitted to the NCBI SRA database (SRA accession PRJNA508306).

### Fatty Acid Methyl Ester Analysis and Microbial Biomass

Phospholipid-derived fatty acids (PLFAs) were extracted from 1 g of sieved, root-free, freeze-dried soil. We used a modified Bligh and Dyer (1959) extraction procedure (White et al., 1979; Guckert et al., 1985) where a single-phase solvent system (chloroform) was modified to include a phosphate buffer. This initially extracts lipids from only viable microorganisms captured at the time of sampling. Lipid extracts were then fractionated on silicic acid columns into neutral, glyco- and polar lipids. Polar lipids were collected and then methylated with 0.2 M methanolic KOH to form fatty acid methyl esters (FAMEs). Purified FAMEs were brought to volume with hexane before injection onto a Varian 3800 FID GC. FAME identification and quantification of each peak was based on retention time data with known standards from Matreya, LLC^®^. The polyenoic unsaturated fatty acids, 18:2ω6 and 18:1ω9c, were considered as fungal biomarkers (Bardgett et al., 1996; Bååth, 2003). Bacterial markers included saturated Gram-positive fatty acids (i15:0, a15:0, i16:0, i17:0, and a17:0), monoenoic and cyclopropane unsaturated Gram-negative fatty acids (18:1ω7c and cy19:0), and general bacterial markers (15:0, 16:1ω7c, and 16:1ω7t) and fungal markers included non arbuscular mycorrhizal fungi (18:2ω6,9 and 18:1ω9) and arbuscular mycorrhizal fungi (AMF)(16:1ω5) (Ekelund et al., 2003; Leckie et al., 2004).

### Enzyme Activities

Potential soil microbial extracellular enzyme activity was assessed using protocols outlined by Saiya-Cork et al. (2002); Grandy et al. (2008), and Wickings and Grandy (2011). The activities of three hydrolytic enzymes, N-acetyl-β-D-glucosaminidase (NAG), β-glucosidase (BG) and acid phosphatase (PHOS) and two oxidative enzymes, phenol oxidase (POX) and peroxidase (PER), were measured. Soil slurries were created from a 1 g soil subsample from each mesocosm and 120 mL sodium acetate buffer (pH 6.5). Hydrolytic enzyme activities were measured on black 96 well plates receiving one of three different substrates and the fluorescent compound methylumbelliferone (MUB). Oxidative enzymes were measured using clear 96 well plates, receiving L-3,4-dihydroxyphenylalanine (L-DOPA) alone for phenol oxidase or L-DOPA plus hydrogen peroxide for peroxidase. Hydrolytic enzyme plates were incubated for 3–4 h and oxidative enzyme plates were incubated for 22–24 h. Hydrolytic enzyme plates were then run at 360 nm excitation and 460 nm emission wavelengths and oxidative enzyme plates at 450 nm absorbance wavelength using a microplate reader (Synergy, BioTek Instruments, Winooski, VT, United States). Potential enzyme activity for each substrate was calculated as nmol of substrate h^-1^ g^-1^ dry soil.

### Carbon and Nitrogen Mineralization

Soils from mesocosms collected in the second year (2 years of burial) were used for estimating potential carbon mineralization. Soils were air-dried the day of collection and remoistened 5 days later to 60% water holding capacity. Soil CO_2_ flux was measured using 30 g of soil in 90 mL serum vials. The serum vials were capped and two headspace measurements were taken, one immediately after capping and one after a pre-determined time to assess accumulation of CO_2_. The incubation time between the initial and second gas measurement was increased progressively from 2 to 24 h over 30 days. Measurements were taken daily for 15 days then at 2–3 day intervals from days 15 to 30. CO_2_ concentrations were determined using a LiCor 820 infrared gas analyzer (LiCor, Lincoln, NE, United States). CO_2_ flux was calculated using the increase in CO_2_ from the initial to the second measurement and by converting ppm CO_2_ to μg C-CO_2_ g^-1^ soil day^-1^. Cumulative CO_2_ over the 30-day incubation period was calculated by consecutively adding the daily flux measurements.

Soil samples collected at the start and at the end of the 30-day incubation period for C mineralization (above) were extracted with 0.05 M K_2_SO_4_ to quantify nitrogen mineralization and net nitrification. Ammonium and nitrate were measured using colorimetric assays in clear 96-well plates, modified from Doane and Horwath (2003) and Sinsabaugh (2010). To obtain potential nitrogen mineralization and nitrification values, the start of incubation values were subtracted from the end of incubation values. Accumulation of ammonium, nitrate and net nitrogen mineralization (ammonium + nitrate) results were reported in μg g^-1^ dry soil day^-1^.

### Statistical Analyses

The overall experimental design was a randomized complete block design with 16 replicate plots and in the end 29 total mesocosms divided across two collection dates. Linear mixed effect models were used to analyze soil invertebrates (macroinvertebrates and mesofauna), microbial biomass through PLFAs and extracellular enzyme activities within the mesocosms. Mesocosm mesh size was treated as the fixed effect and plot and mesocosm were treated as random effects. Measurements after 1 year of burial and 2 years of burial were analyzed separately for these measurements. Root biomass was analyzed separately with a linear mixed effect model with mesocosm mesh size and number of years of burial as fixed effects and plot and mesocosm as random effects. Data normality was determined graphically through histograms and Q–Q plots for residuals. To identify significant mesh size effects, analysis of variance (ANOVA) was used with Satterthwaite’s method to calculate degrees of freedom.

The effects of mesocosm mesh size and burial time on soil bacterial and fungal communities were analyzed via permutational multivariate analysis of variance (PERMANOVA). Bray–Curtis dissimilarities between samples were calculated and non-metric multidimensional scaling (NMDS) was used to visualize differences in the composition of the microbial communities. Invertebrate effects (densities of macroinvertebrates and mesofauna) on the microbial community were visualized using vectors plotting correlations to the ordination. Significance values for the vectors were generated with 999 permutations. The differences in Shannon diversity and relative abundance of each phylum were assessed using linear mixed effect models, where mesocosm mesh size was treated as a fixed effect and plot and mesocosm were treated as random effects. The 2 years of the study were analyzed separately for Shannon diversity and relative abundance of each phylum.

For carbon mineralization, the cumulative and daily measurements were analyzed using repeated measures ANOVA, with mesocosm mesh size and measurement date as between-subject effects and individual mesocosms as within-subject effects. Nitrogen mineralization values were also analyzed using a linear mixed effect model with mesocosm mesh size as a fixed effect and plot and mesocosm as random effects.

Data on soils collected from outside the mesocosms were also analyzed using linear mixed effects models with a fixed effect that included the two mesh sizes and outside as treatments and plot and mesocosm as random effects. This analysis was done in order to distinguish mesocosm effects on soil biota and biological traits with differences for undisturbed soils.

All statistical analyses were performed in R (R Core Team, 2018). The lme4 and lmerTest packages were used for the linear mixed effects models. The phyloseq package was used to aggregate OTU tables. The vegan package was used microbial community analyses (PERMANOVA, Bray–Curtis distances, NDMS, and Shannon diversity). All *P*-values less than 0.05 were considered significant.

## Results

### Soil Fauna Within and Outside Mesocosms

Soil macroinvertebrates included earthworms (Lumbricidae), centipedes (Chilopoda), millipedes (Diplopoda), and beetle larvae (Elateridae and Scarabaeidae) (Table 1). Immature lumbricid earthworms were the dominant macroinvertebrate group. Macroinvertebrate densities were significantly different between treatments as macroinvertebrates were absent from the meso-mesh for both years of the study (1 year: *F*_1,14_ = 48.8, *P* < 0.000001, 2 years: *F*_1,10_ = 8.75, *P* < 0.02; Table 1), confirming that the small mesh size excluded macroinvertebrates. Soil mesofauna were comprised mainly of collembola and mites, with no significant differences in mesofaunal densities between treatments in either year (1 year: *F*_1,10_ = 1.78, *P* = 0.21, 2 years: *F*_1,10_ = 0.894, *P* = 0.37; Table 1), confirming that colonization of the mesocosm soils by mesofauna communities was not impacted by mesh size.

**Table 1:** Fauna densities (number individuals kg^-1^ dry soil, average ± standard error) for mesocosms with macroinvertebrates (macro) and mesocosms excluding macroinvertebrates (meso) and associated ANOVA results.

	1 Year		2 Years	
	Macro	Meso	Macro	Meso
Lumbricidae	1.1 (0.3)	0	1.0 (0.5)	0
Diplopoda	0.3 (0.2)	0	0.2 (0.2)	0
Chilopoda	0.1 (0.1)	0	0	0
Elateridae	0.3 (0.2)	0	0	0
Scarabaeidae	0.1 (0.1)	0	1.1 (0.5)	0
*Total macroinvertebrates*	1.9 (0.3) a	0 b	2.3 (0.9) A	0 B
	*n* = 8	*n* = 8	*n* = 6	*n* = 7
*ANOVA*	*F*_1,14_ = 48.8, *P* < 0.000001		*F*_1,10_ = 8.8, *P* < 0.05	
Collembola	9.6 (2.1)	11.1 (1.9)	13.9 (4.5)	10.8 (2.8)
Oribatida	49.2 (54)	65.0 (14.2)	27.8 (11.4)	18.3 (5.4)
Mesostigmata	12.8 (2.7)	13.0 (1.6)	11.4 (5.5)	11.9 (2.8)
*Total mesofauna*	71.6 (8.3)	89.1 (11.9)	53.2 (17.1)	41.0 (6.9)
	*n* = 7	*n* = 5	*n* = 6	*n* = 7
*ANOVA*	*F*_1,10_ = 1.8, *P* = 0.2		*F*_1,10_ = 0.9, *P* = 0.4	

*Letters denote significant differences between treatments within each year.*

Macroinvertebrate densities inside the mesocosms were significantly lower than outside the mesocosms (1 year: *F*_2,25_ = 18.7, *P* < 0.0001, 2 years: *F*_2,22_ = 21.9, *P* < 0.00001; Supplementary Table 1) where outside mesocosms had 56–62% more macroinvertebrates compared to inside the mesocosms. Similarly, mesofauna densities inside the mesocosms were significantly lower than densities outside the mesocosms (1 year: *F*_2,20_ = 28.5, *P* < 0.00001, 2 years: *F*_2,17.4_ = 21.9, *P* < 0.001; Supplementary Table 1), where outside mesocosm soils had 83–84% more mesofauna. More specifically, for both years of the study, oribatid mites were the least successful colonizers of the mesocosms relative to densities assessed outside the mesocosms (88–90% decrease in oribatid mites inside compared to outside mesocosm) but were the most abundant animal group collected within the mesocosms (Table 1).

### Root Biomass Within Mesocosms

After 1 year of burial, average total root biomass within the mesocosms was 0.81 ± 0.36 or 1027 ± 329 g cubic meter soil^-1^ for macro-mesh and 0.41 ± 0.11 or 522 ± 136 g cubic meter soil^-1^ for meso-mesh mesocosms. After 2 years of burial, average total root biomass within the mesocosms was 0.62 ± 0.17 or 787 ± 219 g cubic meter soil^-1^ for macro-mesh and 0.40 ± 0.050 or 511 ± 63.5 g cubic meter soil^-1^ for meso-mesh mesocosms, respectively. Root biomass within the mesocosms was not significantly different between treatments (*F*_1,24_ = 1.85, *P* = 0.19) or between years (*F*_1,24_ = 0.891, *P* = 0.35) and the treatment-year interaction was also not significant (*F*_1,24_ = 0.526, *P* = 0.48; Table 2). The turfgrass roots growing into the mesocosms extended to the full depth or almost full depth of the mesocosms (10 cm).

**Table 2:** Shannon diversity index (average ± standard error) for bacterial diversity and fungal diversity for mesocosms with macroinvertebrates (macro) and mesocosms excluding macroinvertebrates (meso).

	1 Year		2 Years	
	Macro	Meso	Macro	Meso
16S	5.99 (0.05) a	5.61 (0.06) b	6.14 (0.04)	6.07 (0.06)
ITS	3.43 (0.14)	2.94 (0.23)	3.61 (0.15)	3.48 (0.08)

*Letters denote significant differences between treatments within each year.*

### Microbial Community Composition and Diversity

**FIGURE 1:**
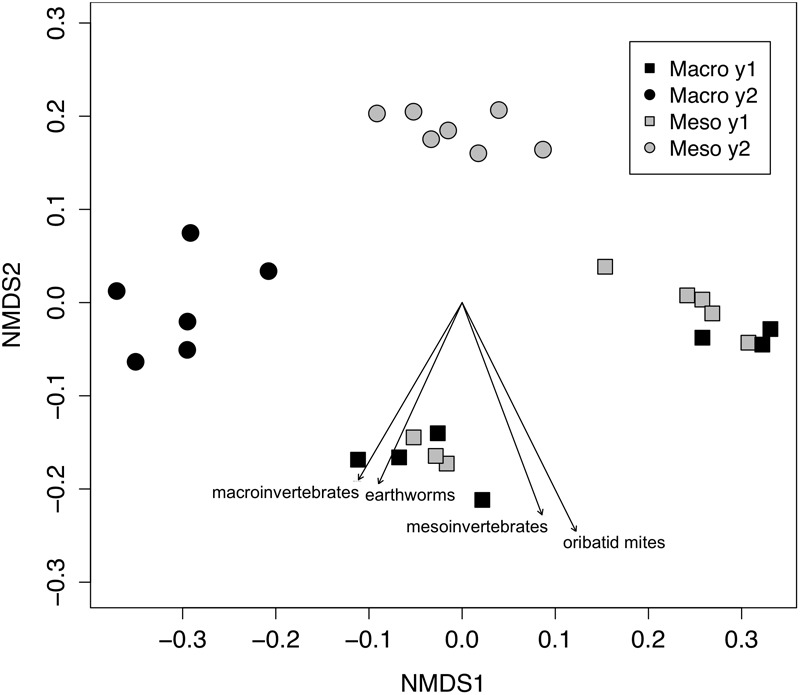
Non-metric multidimensional scaling (NMDS) of bacterial community from 16SrRNA (Bray–Curtis dissimilarities). Macroinvertebrate manipulations resulted in significant shifts in bacterial taxa (PERMANOVA, *P* < 0.01). Vectors indicate significant correlations (*P* < 0.05) between invertebrate densities and microbial community ordination scores for ordination axis two. Vectors include total macroinvertebrate density (macroinvertebrates), earthworm (earthworms), total mesofauna density (mesoinvertebrates) and oribatid mite density (oribatid mites). Non-significant vectors are not shown. Black symbols denote microbial communities from soils permitting macroinvertebrates. Gray symbols denote microbial communities from soils excluding macroinvertebrates. Squares indicate communities from year one and circles from year two.

**FIGURE 2:**
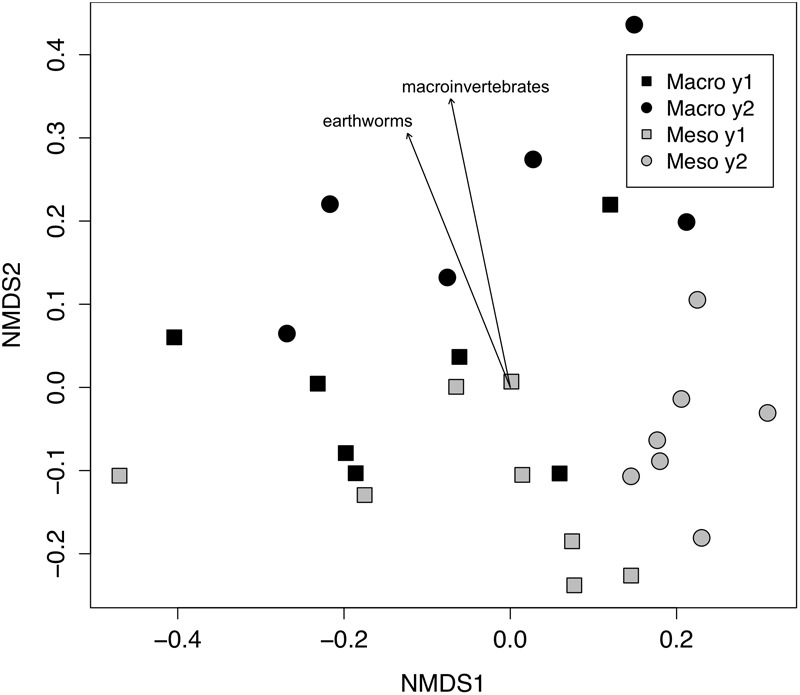
Non-metric multidimensional scaling (NMDS) of fungal community from ITS (Bray–Curtis dissimilarities). Macroinvertebrate manipulations resulted in significant shifts in fungal taxa (PERMANOVA, *P* < 0.01). Vectors indicate significant correlations (*P* < 0.01) between invertebrate densities and microbial community ordination scores for ordination axis two. Vectors include total macroinvertebrate density (macroinvertebrates) and earthworm density (earthworms). Non-significant vectors are not shown. Black symbols denote microbial communities from soils permitting macroinvertebrates. Gray symbols denote microbial communities from soils excluding macroinvertebrates. Squares indicate communities from year one and circles from year two.

Both bacterial and fungal communities (bacterial 16S rRNA gene and fungal ITS) within mesocosms differed significantly between treatments (*P* < 0.01; Figure 1, 2). Though the differences in the communities were not significant between years (16S: *P* = 0.63, ITS: *P* = 0.81), the treatment by time interaction was significant for the bacterial community (16S: *P* < 0.05, ITS: *P* = 0.11). NMDS ordination also revealed relationships between soil invertebrates and microbial community structure. For the bacterial community ordination, the stress value was 0.0724 and the non-metric fit R^2^ was 0.995. For the fungal community ordination, the stress value was 0.134 and the non-metric R^2^ 0.982. Invertebrate community correlations to the bacterial community ordination were significant for total macroinvertebrate density, earthworm density, total mesofauna density and oribatid mite densities (*P* < 0.05, Figure 1). Invertebrate community correlations to the fungal community ordination were significant for total macroinvertebrate densities and earthworm densities (*P* < 0.01, Figure 2).

Shannon diversity of the bacterial community was significantly higher when macroinvertebrates were present after 1 year of burial (*F*_1,5_ = 39.7, *P* < 0.01; Table 2) but this difference was not significant after 2 years of burial (*F*_1,10_ = 1.21, *P* = 0.30, Table 2). Diversity of the fungal community was not significantly altered by treatment in either year (1 year: *F*_1,12_ = 3.13, *P* = 0.10; 2 years: *F*_1,10_ = 0.596, *P* = 0.46; Table 2). Microbial community composition measurements from outside the mesocosms are included in the supplement (Supplementary Figures 1, 2 and Supplementary Table 2).

The most abundant bacterial phyla identified were Acidobacteria, Actinobacteria, Bacteroidetes, Firmicutes, Planctomycetes, Proteobacteria, and Verrucomicrobia. Relative abundance of Actinobacteria (macro: 15.9 ± 1.6, meso: 23.3 ± 1.6) and Firmicutes (macro: 1.0 ± 0.1, meso: 3.7 ± 0.3) was significantly lower with macroinvertebrates after 1 year of burial (*F*_1,13_ = 10.4, *P* < 0.01; *F*_1,13_ = 70.0, *P* < 0.00001; Supplementary Table 2), whereas the relative abundance of Bacteroidetes (macro: 12.7 ± 1.7, meso: 6.4 ± 0.4) and Verruomicrobia (macro: 8.7 ± 0.4, meso: 6.7 ± 0.4) was significantly higher (*F*_1,13_ = 19.5, *P* < 0.001; *F*_1,13_ = 11.9, *P* < 0.01; Supplementary Table 2). After 2 years of burial, the pattern for the relative abundance of Actinobacteria (macro: 9.0 ± 0.9, meso: 13.0 ± 1.2) and Firmicutes (macro: 1.4 ± 0.3, meso: 3.5 ± 0.5) (*F*_1,10_ = 12.9, *P* < 0.01; *F*_1,10_ = 19.8, *P* < 0.01; Supplementary Table 2) remained the same and was also evident in Planctomycetes (macro: 6.6 ± 0.2, meso: 4.4 ± 0.4) (*F*_1,10_ = 21.4, *P* < 0.001; Supplementary Table 2).

The most abundant fungal phyla were Ascomycota, Basidiomycota, and Zygomycota. There were no significant differences in relative abundance after 1 year of burial; however, the relative abundance of Zygomycota (macro: 17.3 ± 1.5, meso: 32.3 ± 3.5) was significantly lower with macroinvertebrates after 2 years of burial (*F*_1,10_ = 13.3, *P* < 0.01; Supplementary Table 2).

### Microbial Biomass (PLFAs)

**FIGURE 3:**
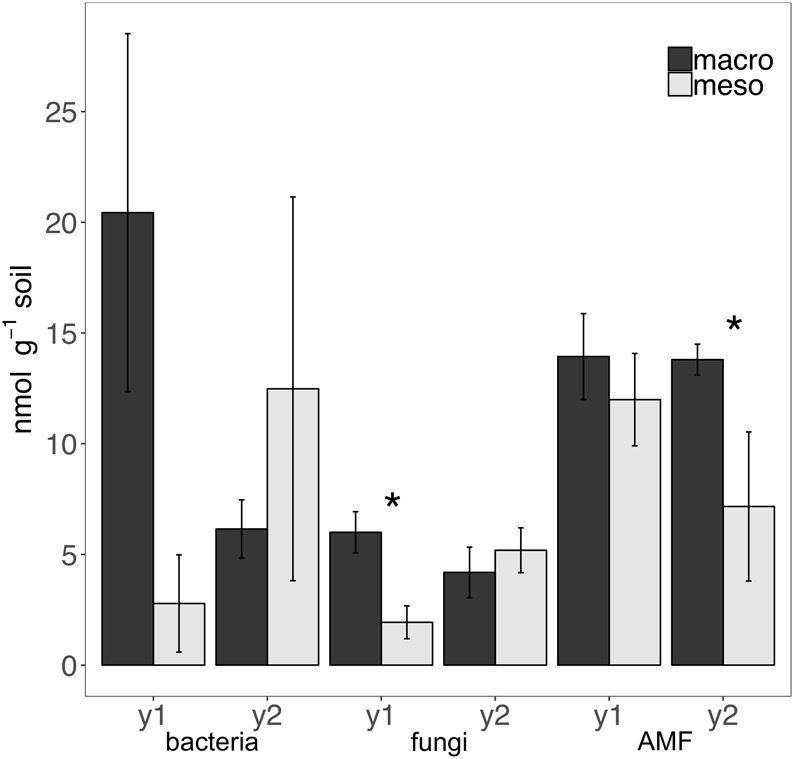
Microbial biomass based on phospholipid fatty acids (PLFAs) (nmol g^-1^ soil, average ± standard error) in years 1 (y1) and 2 (y2) for total bacteria, total non-AMF fungi (fungi) and AMF. Mesocosms with macroinvertebrates (macro) are represented in dark gray and mesocosms excluding macroinvertebrates (meso) are in light gray. Asterisks denote significant differences between mesocosm treatments within years (*P* < 0.05).

Total bacterial biomass based upon PLFAs was not significantly different between treatments for either year (1 year: *F*_1,6_ = 5.44, *P* = 0.56; 2 years: *F*_1,7_ = 1.09, *P* = 0.33; Figure 3), although there was a trend toward higher bacterial biomass in year 1 with macroinvertebrates present (Figure 3). Total non-AMF fungal biomass from PLFAs was significantly higher when macroinvertebrates were present for year 1 (+68%) (*F*_1,6_ = 8.671, *P* < 0.05; Figure 3) but not for year 2 (*F*_1,7_ = 0.34, *P* = 0.58). The opposite pattern was observed in AMF biomass, which was significantly higher in mesocosms with macroinvertebrates after 2 years (+48%) (*F*_1,8_ = 5.61,1, *P* < 0.05; Figure 3). Total bacterial and non-AMF fungal biomass from PLFAs was significantly higher when macroinvertebrates were present for year 1 (+82%) (*F*_1,6_ = 6.35, *P* < 0.05; Figure 3) but not for year 2 (*F*_1,8_ = 0.81, *P* = 0.40). Macroinvertebrates did not significantly affect the fungal to bacterial ratio in either year (1 year: *F*_1,7_ = 0.0053, *P* = 0.94; year 2: *F*_1,8_ = 51.64, *P* = 0.24). Complete lipid marker profiles are summarized in Table 3.

**Table 3:** Phospholipid fatty acid (PLFA) lipid biomarkers (nmol g^-1^ soil, average ± standard error) for bacteria, fungi, and AM fungi across both years for mesocosms with macroinvertebrates (macro) and mesocosms excluding macroinvertebrates (meso).

	1 Year		2 Years	
	Macro	Meso	Macro	Meso
**Bacterial markers**				
i15:0	7.30 (2.81)	1.41 (1.40)	3.77 (0.71)	5.28 (3.35)
a15:0	4.66 (1.74)	0.89 (0.90)	1.33 (0.66)	2.53 (1.95)
15:0	0.20 (0.09)	0.16 (0.16)	0.01 (0.09)	0.09
i16:0	2.46 (1.12)	0	0	1.00 (1.03)
16:1w7c	2.36 (1.06)	0	0.33 (0.31)	1.51 (0.91)
i17:0	1.73 (0.79)	0	0	0.993
a17:0	1.01 (0.46)	0	0	0.490
18:1w7c	0.02 (0.01)	0	0	8 × 10^-3^ (8 × 10^-4^)
cy19:0	0.69 (0.07)	0.32 (0.04)	0.62 (0.11)	0.57 (0.02)
**Fungal markers**				
18:2w6,9	2.24 (0.22)	1.26 (0.25)	2.32 (0.31)	1.82 (0.02)
18:1w9	3.76 (0.81)	0.67 (0.67)	1.87 (0.87)	3.37 (1.02)
**AMF marker**				
16:1w5	13.94 (1.94)	12.00 (2.09)	13.80 (0.70)	7.17 (3.37)

### Enzymes

**FIGURE 4:**
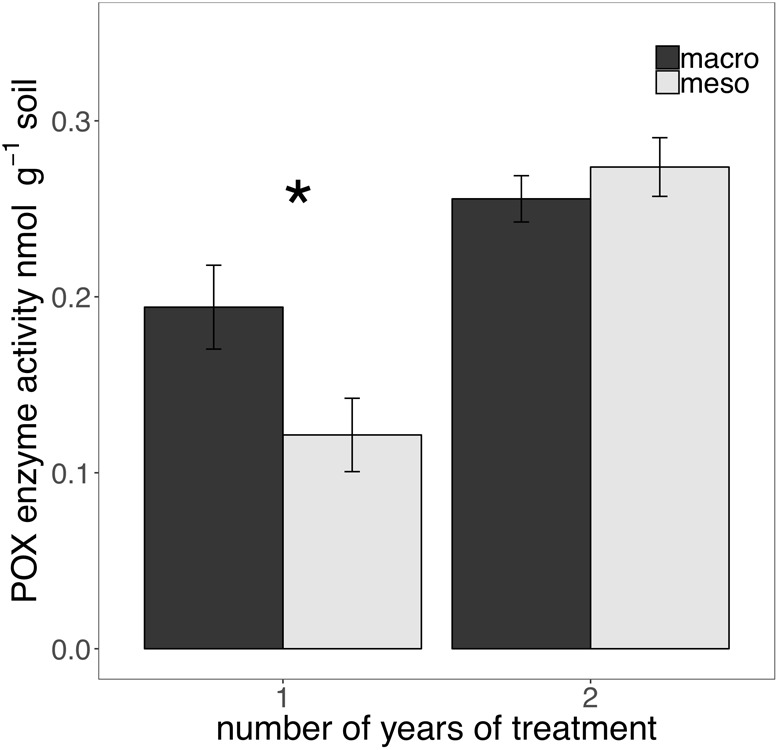
Potential extracellular phenol oxidase (POX) activity (nmol g^-1^ soil, average ± standard error) in years 1 and 2. Mesocosms with macroinvertebrates (macro) are represented in dark gray and mesocosms excluding macroinvertebrates (meso) are in light gray. Asterisks denote significant differences between mesocosm treatments within years (*P* < 0.05).

**FIGURE 5:**
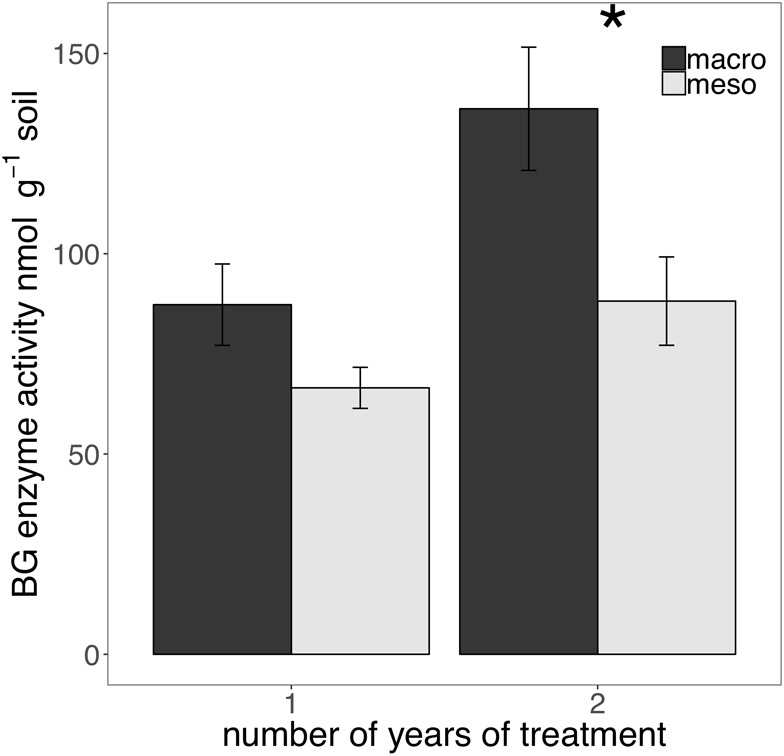
Potential extracellular β-glucosidase (BG) activity (nmol g^-1^ soil, average ± standard error) in years 1 and 2. Mesocosms with macroinvertebrates (macro) are represented in dark gray and mesocosms excluding macroinvertebrates (meso) are in light gray. Asterisks denote significant differences between mesocosm treatments within years (*P* < 0.05).

The presence of macroinvertebrates increased the activity of phenol oxidase (POX) by 37.5% (*F*_1,13_ = 5.38, *P* < 0.05; Figure 4) after 1 year of burial and β-glucosidase (BG) by 35.3% (*F*_1,12_ = 6.82, *P* < 0.05; Figure 5) after 2 years. Other measured enzymes, NAG, PHOS, and PER showed no differences across treatments in either year of the study (averages for each year reported in Supplementary Table 1).

### Carbon and Nitrogen Mineralization

**FIGURE 6:**
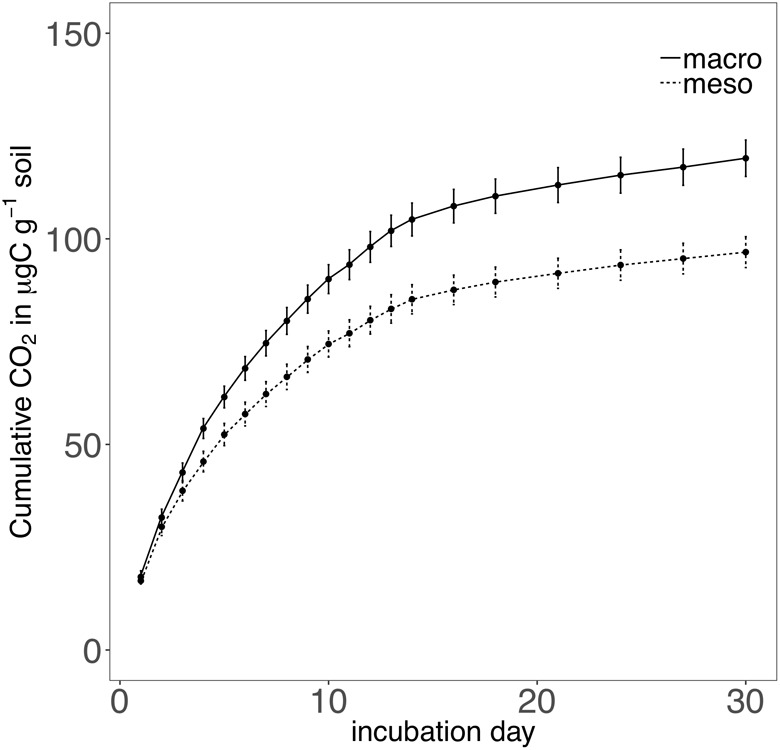
Cumulative CO_2_ (μg carbon g^-1^ soil) over a 30 day incubation from mesocosm soils recovered following 2 years of burial. Mesocosms with macroinvertebrates (macro) are represented with a solid line and mesocosms excluding macroinvertebrates (meso) are represented with a dashed line. Asterisks denote significant differences between mesocosm treatments (*P* < 0.05).

**FIGURE 7:**
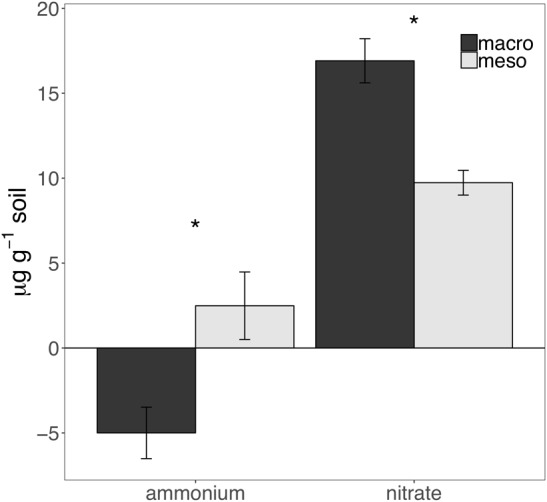
Ammonium and nitrate mineralization (μg g^-1^ soil, average ± standard error) following 30 days of incubation of mesocosm soils collected after 2 years of burial. Mesocosms with macroinvertebrates (macro) are represented in dark gray and mesocosms excluding macroinvertebrates (meso) are in light gray. Asterisks denote significant differences between mesocosm treatments (*P* < 0.05).

Cumulative carbon mineralization potential measured after 2 years differed between the two treatments (*F*_1,19_ = 10.9, *P* < 0.01; Figure 6 and Supplementary Table 3) and was significantly higher when macroinvertebrates were included (+19%). Net ammonium and net nitrate were both affected by mesocosm treatment: net ammonium accumulation was lower (-150%) (*F*_1,7_ = 24.4, *P* < 0.01; Figure 7 and Supplementary Table 3) and net nitrification was higher (+30%) (*F*_1,11_ = 25.1, *P* < 0.01; Figure 7 and Supplementary Table 3) when macroinvertebrates were included. However, potential net nitrogen mineralization (ammonium plus nitrate) was not significantly between the two fauna treatments 2 years after burial (*F*_1,6_ = 0.181, *P* = 0.7).

## Discussion

Our findings indicate that macroinvertebrates can significantly affect microbial community composition and that they stimulate microbial biomass and activity within the rhizosphere. Soil macroinvertebrates increased bacterial diversity and stimulated microbial biomass, phenol oxidase and glucosidase activities along with potential carbon mineralization and nitrification. However, contrary to our hypothesis, macroinvertebrates did not cause a decrease in the fungal to bacterial ratio. Instead, fungal biomass increased significantly at least after the first year of the study in the presence of macroinvertebrates. Moreover, many of the observed treatment responses that were significant after the first year were not observed after 2 years.

Some of the differences in treatment responses between the first and second year are likely a result of changing physical and chemical conditions within the mesocosms. The sieving and defaunation process used to eliminate soil animals prior to the study likely resulted in a pulse of labile organic matter as pre-existing macroaggregates were disturbed or destroyed (Six et al., 2000; Denef et al., 2001); however, such an effect on organic matter availability to decomposers should be relatively short-lived and probably minimal after a full year of incubation. The introduction of new grass roots and colonization by soil invertebrates and microbial communities would be expected to result in transient changes in biotic communities and activity during the first growing season and may explain some of the treatments effects observed in year 1. Additionally, soil invertebrate densities within the mesocosms were lower compared to outside the mesocosms. The length of the experiment (2 years) highlights how slowly invertebrates recolonize soils. This is supported by previous work showing both slow recovery of soil by invertebrates following disturbance (Adl et al., 2006) along with taxon- and system-dependent differences in soil animal colonization (Cole et al., 2006). Our findings provide insight into the role of soil animals in shaping microbial processes as both microbes and invertebrates colonize new habitats or existing soil following disturbances. We suggest that these initial responses to the presence of macroinvertebrates represent a reorganization phase of soil ecosystem development, and were perhaps more apparent during the first year of the study due to the initial colonization of the soil by roots and invertebrates and reestablishment of microbial communities.

Our mesocosm soils were primarily populated by a mixture of earthworms, herbivorous beetle larvae and predatory and detritivorous myriapods. Previous studies have shown that macroinvertebrates elicit mixed microbial responses that can vary even at the species level. For example, earthworms were found to increase microbial biomass, whereas millipedes decreased biomass and their co-occurrence led to an increase in biomass (Seeber et al., 2006). Additionally, different species of native and invasive earthworms had dissimilar effects on microbial biomass (Zhang et al., 2000; Scheu et al., 2002; Chang et al., 2017). The abundance of earthworms in our macro-mesh mesocosms was moderately high (approximately 180 individuals per m^2^ on average across both years) compared to other grasslands (e.g., Gastine et al., 2003; Xu et al., 2013) and nearby forested areas (e.g., Dempsey et al., 2011), although lower (56–62%) compared to undisturbed soils adjacent to the mesocosms. The variability in microbial response observed among studies is likely due to differences in behavior and ecological function such as SOM mixing, microbial grazing intensity and patterns of fecal/waste deposition among different macroinvertebrate species (Ineson and Anderson, 1985; Hassall et al., 1987; Devliegher and Verstraete, 1995, 1997; Brown et al., 2000). Thus, the impact of macroinvertebrates on microbial biomass likely depends upon macroinvertebrate abundance and community composition. Our findings indicate that despite large differences in ecological function and potential interactions types among the macroinvertebrates occurring under natural conditions within our mesocosms their presence had an overall stimulatory effect on microbial biomass.

The observed increase in microbial biomass was driven primarily by an increase in fungal biomass, specifically non-AMF fungi in year 1 and AMF in year 2. This finding was surprising given that many previous studies have shown that macroinvertebrates decrease fungal biomass (Crowther et al., 2011a; Dempsey et al., 2011 but see Dempsey et al., 2013). Fungi are an important food source for earthworms and other macroinvertebrates (Bonkowski et al., 2000; Brown et al., 2004; Pollierer et al., 2007) and therefore it is logical that macroinvertebrate activity would result in a decline in fungal biomass. However, effects on fungal biomass have been shown to vary with grazer identity and grazing intensity. For example, Crowther and A’Bear (2012) concluded that high intensity grazing by larger invertebrates decreases microbial biomass, whereas low intensity grazing by smaller mesoinvertebrates and other mesofauna can increase microbial biomass. Increases in biomass due to invertebrate grazing have also been attributed to compensatory growth (Lussenhop, 1992). The density of macroinvertebrates in our mesocosms was lower compared to the typical densities observed outside of our mesocosms, suggesting that grazing intensity by macroinvertebrates in our soils may have been relatively low, resulting in stimulation rather than suppression of fungal biomass. The presence of earthworms has also been shown to increase AMF colonization rates (Zarea et al., 2009). Our observed increased in AMF in year 2 in the presence of macroinvertebrates supports this finding; however, we were unable to confirm whether the increase in AMF in soil was associated with an increase in AMF root colonization.

Under natural conditions, grazing is only one of the many macroinvertebrate activities that fungi are exposed to. For instance, bioengineering and litter-soil mixing by macroinvertebrates can improve soil habitat and resource distribution for microbes (Lavelle and Spain, 2001; Coleman et al., 2004). Similarly, many macroinvertebrates are capable of fungal spore dispersal through gut passage and fecal deposition, or by the passive transport of fungal spores on their exoskeletons or cuticles (McIlveen and Cole, 1976; Rabatin and Stinner, 1985; Gange, 1993; Moody et al., 1996; Lilleskov and Bruns, 2005). Our results suggest that fungal grazing either had a stimulatory or minor impact on fungal biomass and that its effects on the microbial community were likely tempered or outweighed by other factors such as improved resource distribution or fungal spore dispersal. Furthermore, the increase in non-AMF fungal biomass was only observed in year 1 and may be due to the introduction of fungi to the mesocosms by macroinvertebrates during the initial colonization phase after 1 year of burial. The disappearance of this effect in year 2 was driven not by a decrease in fungal biomass in the presence of macroinvertebrates, but instead by an increase in their absence. This supports evidence that soil animals do in fact play an important role in fungal spore dispersal (McIlveen and Cole, 1976; Rabatin and Stinner, 1985; Gange, 1993; Moody et al., 1996; Lilleskov and Bruns, 2005) but our results further indicate that fauna across all size classes are capable of contributing to this process over different timescales. Thus the rate of fungal colonization of new resource inputs to soil may depend upon soil animal community composition.

One of the most striking findings of our study was the strong disconnect between microbial biomass and community composition responses to macroinvertebrates. For instance, the increase in fungal biomass observed in year 1 was not accompanied by a change in the relative abundance of any individual fungal phyla, indicating that the initial fungal response to macroinvertebrates was community-wide. However, it is also possible that the fungal taxa responsible for the observed shifts in fungal biomass were not captured by the primers used for ITS sequencing as some fungal taxa are excluded (e.g., Glomeromycota, Schoch et al., 2012). In contrast, we found that macroinvertebrates caused an increase in bacterial diversity and shifts in the relative abundance of the phyla Bacteriodetes, Verrucomicrobia, Actinobacteria, and Firmicutes. Similarly, macroinvertebrates caused a relative decrease in the fungal phylum Zygomycota in year 2. Neither of these taxon-level shifts in bacteria or fungi was associated with notable changes in total bacterial or fungal biomass. One mechanism that may explain this response is the modification of microbial communities upon gut passage and fecal material deposition, which has been shown to alter bacterial community composition (Nechitaylo et al., 2010). For instance, Bacteroidetes is recognized as an important phylum in the digestive tracts of invertebrates (Van Borm et al., 2002; Egert et al., 2003; Schmitt-Wagner et al., 2003; Nechitaylo et al., 2010) and has been shown to increase in soils in response to earthworm additions (Bernard et al., 2012).

Fungus-grazing invertebrates may also be capable of altering fungal community composition through selective grazing on distinct fungal taxa (Klironomos and Kendrick, 1996; Maraun et al., 2003), which may explain the decline in only Zygomycota in year 2 of the study. However, the community-wide increase in total fungal biomass observed in year 1 may reflect the finding that macroinvertebrates show lower selectivity than microinvertebrates, such as nematodes and protozoans, when grazing on fungi (Maraun et al., 2003). Despite these and other studies investigating species-level interactions between microbes and soil invertebrates, most studies on microbial responses to soil animals under field conditions have employed coarse levels of microbial taxonomic resolution. Our study is one of only a handful studies to use next generation sequencing to investigate the impacts of macroinvertebrates on soil microbial communities and demonstrates that microbial responses can emerge at different taxonomic levels (Crowther et al., 2015): some effects (e.g. dispersal of fungal spores) manifest uniformly across taxa, while others (e.g., intensive fungivory) may occur at finer taxonomic levels and can only be captured using sequencing and other molecular approaches.

Changes in microbial biomass and community composition are commonly associated with distinct shifts in microbial activity (Maraun and Scheu, 1996; Groffman et al., 2004; Hedde et al., 2007) and our findings indicate that macrofauna modify both taxonomic and functional aspects of soil microbial communities within the rhizosphere. Specifically, macrofauna stimulated phenol oxidase activity in year 1 and glucosidase activity in year 2. Beta glucosidase is important for the breakdown of labile carbon compounds and is often used as a general indicator for SOM cycling (Stott et al., 2010), while phenol oxidase plays a role in lignin degradation and is responsive to shifts in plant and microbial communities (Sinsabaugh, 2010). This finding is supported by previous work showing that invertebrates often stimulate microbial C and N cycling activities (Ineson and Anderson, 1985; Hassall et al., 1987; Wickings and Grandy, 2011). The increase in phenol oxidase activity in year 1 may have been driven by the community-wide increase in fungal biomass, as fungi are known to produce lignin-degrading enzymes (Fioretto et al., 2000; Romani et al., 2006; Harner et al., 2009; Sinsabaugh, 2010). Phenol oxidase activity may have also been stimulated by the consumption and gut passage of fine roots by macroinvertebrates during the first year of the study (Fisk et al., 2004). The functional consequences of phylum-level changes in the microbial community are more difficult to interpret. However, there is some evidence that suggests that Bacteroidetes, classified as copiotrophic (Fierer et al., 2007), are associated with increased carbon availability and carbon mineralization rates. This explanation aligns with the stimulation of glucosidase activity and carbon mineralization observed in year 2 of the study. The sieving and defaunation process used to establish our treatments likely created a pulse of labile organic matter within our mesocosm soils. We would expect this to elicit stimulatory responses in hydrolytic microbial enzyme activities (Fontaine et al., 2003) in the first year of the experiment, yet we observed an increase in phenol oxidase activity only. The pulse of labile organic matter was likely not captured in sampling 1 year after burial of the mesocosms and potentially could have been measured if samples had been taken immediately after the installation of the mesocosms and in the weeks and months following. Macroinvertebrate activity also caused notable responses in nitrogen cycling by microbes: increased immobilization of ammonium and stimulation of net nitrification, which ultimately led to no net effect on net nitrogen mineralization. Higher nitrification could reflect an increased abundance of nitrifier species and nitrification rates in casts and burrows of lumbricid earthworms, as has been observed in agricultural soils by Parkin and Berry (1994, 1999), however, our sampling approach prevented us from distinguishing species-level responses in the microbial communities.

## Conclusion

Our findings highlight the potentially important role of macroinvertebrate communities in shaping the composition and activity soil microbial communities within the rhizosphere. Despite evidence in previous studies for both positive and negative effects of macrofauna on microbial biomass and activity, our findings suggest that under natural conditions, with diverse invertebrate communities, macroinvertebrates stimulate microbial biomass and processes. Such stimulatory responses may stem from diverse animal-microbe interaction types such as low-intensity grazing, microbial dispersal and changes in microbial resource quality or availability. We also observed macroinvertebrate-driven shifts in microbial community composition that extend out to impact carbon and nutrient cycling. We remain cautious in interpreting our findings beyond the 2-year scope of our study and acknowledge that the treatment effects observed likely reflect a community establishment and colonization phase for both microbes and invertebrates. Longer-term experiments will be required to fully capture the role of mixed macroinvertebrate communities in shaping microbial dynamics under natural conditions. Future studies on fauna-microbe interactions should also continue to incorporate sequencing and other molecular approaches to fully elucidate both community-wide and taxon-specific changes in the microbial communities and their consequences for soil ecosystem processes such as SOM formation in response to soil invertebrates.

## Author Contributions

NB and KW designed the experiments and wrote the first draft of the manuscript. NB performed the experiments under the supervision of KW and JK-K, except for PLFA analyses which were performed under the direction of SF. NB analyzed the data. All authors revised the text of the manuscript.

## Conflict of Interest Statement

The authors declare that the research was conducted in the absence of any commercial or financial relationships that could be construed as a potential conflict of interest.
