# Supplementary material for: Soil Macroinvertebrate Presence Alters Microbial Community Composition and Activity in the Rhizosphere
Source: Front Microbiol. 2019 Feb 22;10:256. doi: 10.3389/fmicb.2019.00256 (PMC6395791; doi:10.3389/fmicb.2019.00256)
Supplement: Supplementary file 1 [file Table_1.docx]

Supplementary Material

Soil Macroinvertebrate Presence Alters Microbial Community Composition and Activity in the Rhizosphere

Natalie Bray^1*^, Jenny Kao-Kniffin^2^, Serita D. Frey^3^, Timothy Fahey^4^, Kyle Wickings^1^

^1^ Department of Entomology, Cornell AgriTech, Cornell University, Geneva, NY, USA

^2^ School of Integrative Plant Science, Cornell University, Ithaca, NY, USA

^3^ Department of Natural Resources and the Environment, University of New Hampshire, Durham, NH, USA

^4^ Department of Natural Resources, Cornell University, Ithaca, NY, USA

*** Correspondence:**Kyle Wickings
kgw37@cornell.edu

## Supplementary Figures

**
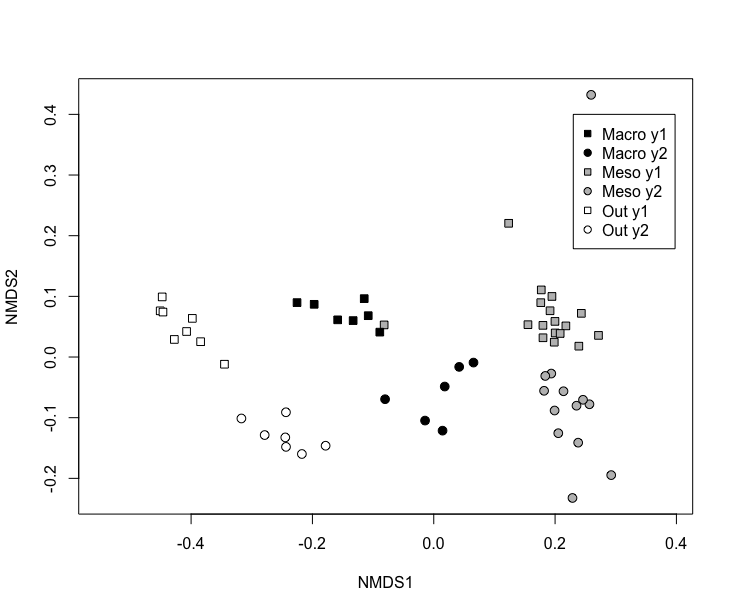
**

**Supplementary Figure 1:** Non-metric multidimensional scaling **(**NMDS) of bacterial community from 16SrRNA (Bray-Curtis dissimilarities). Macroinvertebrate manipulations resulted in significant shifts in bacterial taxa (PERMANOVA, *P* < 0.01). Black symbols denote microbial communities from soils permitting macroinvertebrates. Gray symbols denote bacterial communities from soils excluding macroinvertebrates. Open symbols denote bacterial communities from soils adjacent to mesocosm. Squares indicate communities from year one and circles from year two.

**
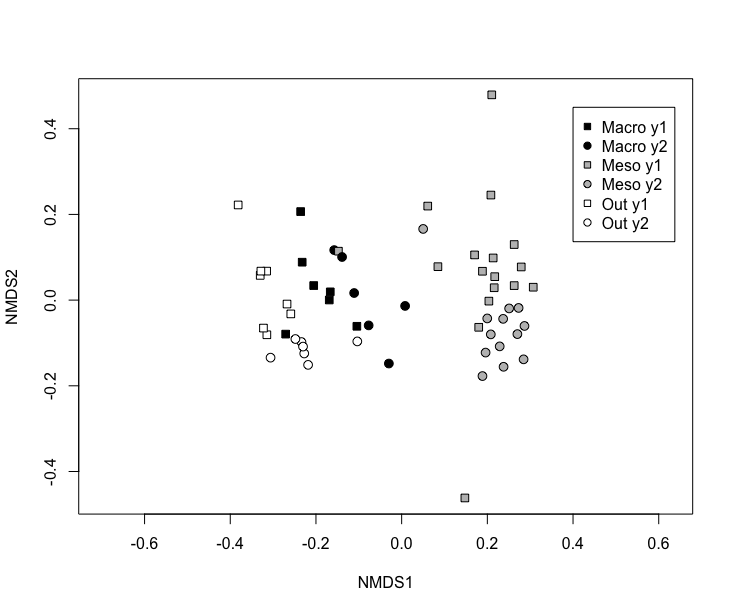
**

**Supplementary Figure 2:** Non-metric multidimensional scaling **(**NMDS) of fungal community from ITS (Bray-Curtis dissimilarities). Macroinvertebrate manipulations resulted in significant shifts in fungal taxa (PERMANOVA, *P* < 0.01). Black symbols denote fungal communities from soils permitting macroinvertebrates. Gray symbols denote fungal communities from soils excluding macroinvertebrates. Open symbols denote fungal communities from soils adjacent to mesocosm. Squares indicate communities from year one and circles from year two.

## Supplementary Tables

**Supplementary Table 1**: Fauna densities (number individuals kg^-1^ soil), and enzyme activities (nmol h^-1^ g^-1^ soil, average +/- standard error) from soils adjacent to mesocosms

|  | 1 year | 2 years |
| --- | --- | --- |
| **Soil fauna** [individuals kg^-1^ soil] | | |
| Lumbricidae | 4.9 (0.8) | 5.1 (0.5) |
| Diplopoda | 0 | 0 |
| Chilopoda | 0 | 0 |
| Elateridae | 0 | 0 |
| Scarabaeidae | 0 | 0 |
| *Total macroinvertebrates* | 4.9 (0.8) | 5.1 (0.5) |
| Collembola | 13.6 (2.3) | 39.0 (10.0) |
| Oribatida | 477.8 (60.5) | 230.6 (41.9) |
| Mesostigmata | 26.9 (6.3) | 19.5 (5.5) |
| *Total mesofauna* | 518.2 (56.6) | 289.1 (51.6) |
| **Enzymes** [nmol h^-1^ g^-1^ soil] | | |
| N-acetyl-glucosaminidase | 86.8 (4.5) | 74.5 (4.2) |
| β-glucosidase | 183.4 (13) | 158.0 (9.4) |
| Acid phosphatase | 532.1 (26.8) | 605.3 (26.2) |
| Phenol oxidase | 0.2 (0.01) | 0.31 (0.02) |
| Peroxidase | 0.57 (0.01) | 0.72 (0.03) |

**Supplementary Table 2:** Relative abundance (%) of microbial phyla inside and outside mesocosms 16S and ITS

|  | 1 year | | | 2 years | | |
| --- | --- | --- | --- | --- | --- | --- |
|  | **Macro** | **Meso** | **Out** | **Macro** | **Meso** | **Out** |
| *16S bacteria* | | | | | | |
| Actinobacteria | 15.9 (1.6) | 23.2 (1.6) | 12.1 (0.9) | 9.0  (0.9) | 13.0 (1.2) | 12.5 (0.7) |
| Proteobacteria | 31.8 (0.6) | 32.3 (0.4) | 30.7 (0.8) | 31.8 (0.7) | 29.8 (0.8) | 29.2 (0.6) |
| Firmicutes | 1.0  (0.1) | 3.7  (0.3) | 0.52 (0.1) | 1.4 (0.3) | 3.5 (0.5) | 2.1 (0.3) |
| Acidobacteria | 15.8 (0.6) | 13.5 (0.9) | 24.9 (1.3) | 23.5 (1.4) | 22.3 (0.8) | 19.3 (0.8) |
| Bacteroidetes | 12.7 (1.7) | 6.4 (0.4) | 4.9 (0.5) | 8.1 (0.7) | 6.7 (0.8) | 7.1 (0.4) |
| Planctomycetes | 5.9 (0.3) | 4.8 (0.3) | 7.3 (0.3) | 6.6 (0.2) | 4.4 (0.4) | 7.2 (0.7) |
| Verrucomicrobia | 8.7 (0.4) | 6.7 (0.4) | 9.4 (0.4) | 7.7 (5.9) | 6.3 (0.5) | 7.9 (0.4) |
| *ITS fungi* | | | | | | |
| Ascomycota | 39.2 (3.9) | 35.7 (3.9) | 67.6 (4.4) | 54.5 (4.4) | 49.5 (3.7) | 78.4 (2.9) |
| Basidiomycota | 10.6 (1.8) | 11.2 (1.9) | 8.68 (2.5) | 6.8  (0.7) | 12.2 (2.3) | 7.02 (1.8) |
| Zygomycota | 27.2 (3.2) | 26.7 (4.1) | 5.2  (1.0) | 17.3 (1.5) | 32.3 (3.5) | 3.0 (0.4) |

**Supplementary Table 3**: Ammonium, nitrate and net nitrogen mineralization (μg N g^-1^ soil) and cumulative CO_2_ (μg C g^-1^ soil, average +/- standard error) over 30 day incubation from soils adjacent to mesocosm

|  | 2 years |
| --- | --- |
| **N mineralization over 30 days** [μg g^-1^ soil] | |
| Ammonium | -7.06 (2.5) |
| Nitrate | 20.85 (0.2) |
| Net N | 13.79 (2.4) |
| **Cumulative carbon mineralization per day** [μgC g^-1^ soil] | |
| 1 | 21.9 (1.6) |
| 2 | 42.5 (2.0) |
| 3 | 56.7 (6.8) |
| 4 | 73.5 (7.8) |
| 5 | 85.8 (7.1) |
| 6 | 96.4 (7.7) |
| 7 | 104.5 (7.1) |
| 8 | 112.1 (7.0) |
| 9 | 119.4 (6.5) |
| 10 | 125.5 (6.4) |
| 11 | 130.4 (6.0) |
| 12 | 135.9 (5.8) |
| 13 | 141 (5.6) |
| 14 | 145.5 (5.7) |
| 16 | 150.1 (5.4) |
| 18 | 153.5 (5.4) |
| 21 | 156.9 (5.0) |
| 24 | 159.7 (5.1) |
| 27 | 162.4 (5.0) |
| 30 | 165.0 (5.0) |
